# Brain connectivity patterns associated with individual differences in the access to experience-near personal semantics: a resting-state fMRI study

**DOI:** 10.3758/s13415-023-01149-6

**Published:** 2024-01-10

**Authors:** Alice Teghil, Maddalena Boccia

**Affiliations:** 1 Department of Psychology, “Sapienza” University of Rome, Via dei Marsi, 78, 00185 Rome, Italy; 2 Cognitive and Motor Rehabilitation and Neuroimaging Unit, IRCCS Fondazione Santa Lucia, Rome, Italy

**Keywords:** Autobiographical memory, Autobiographical fluency, Intrinsic functional connectivity

## Abstract

It has been proposed that a continuum of specificity exists between episodic and semantic autobiographical memory. Personal semantics have been theorized to situate intermediately on this continuum, with more “experience-near” personal semantics (enPS) closer to the episodic end. We used individual differences in behavior as a model to investigate brain networks associated with the access to episodic autobiographical (EAM) and enPS information, assessing the relation between performance in the EAM and enPS conditions of the Autobiographical Fluency Task (AFT) and intrinsic brain connectivity. Results of an intrinsic connectivity contrast analysis showed that the global connectivity of two clusters in the left and right posterior cingulate cortex (PCC) was predicted by performance in the enPS conditions. Moreover, enPS scores predicted the connectivity strength of the right PCC with the bilateral anterior hippocampus (aHC), anterior middle temporal gyrus (aMTG) and medial orbitofrontal cortex, and the left aMTG and PCC. enPS scores also predicted the connectivity strength of the left PCC with the bilateral HC and MTG. The network highlighted involves parts of the core and of the dorsal medial subsystems of the Default Mode Network, in line with the proposal that enPS represents an intermediate entity between episodic and semantic memory.

**Supplementary Information:**

The online version contains supplementary material available at 10.3758/s13415-023-01149-6.

## Introduction

The term autobiographical memory (AM) broadly refers to the ability to recollect events and facts concerning one’s own life (Palombo, Sheldon, & Levine, 2018). Autobiographical memory is known to rely on a complex set of cognitive abilities, including domain-general (e.g. search, inhibition) and domain-specific processes (e.g. spatial, self-referential processing) (Palombo, Sheldon, & Levine, 2018) and plays a key role in maintaining the continuity of the self across time (Prebble et al., 2013).

Both a semantic and an episodic component can be identified within the AM system: semantic autobiographical memory (also referred to as personal semantics, PS) involves a representation of personal information and general knowledge related to the self, including autobiographical facts, such as names of relative and friends, the address of one’s house, knowledge of one’s own personality traits and roles, and repeated events (Levine et al., 2004; Renoult et al., 2012; Grilli & Verfaellie, 2014). Episodic autobiographical memory (EAM), instead, involves the representation of specific personal past events characterized by a definite spatial and temporal context, which entails the conscious recollection and reexperiencing of such events in their details and the awareness of the self across time, in what has been defined a “mental time travel” (Tulving, 2002; Levine et al., 2002).

It has been proposed that a continuum of specificity exists between episodic and semantic memory in the autobiographical domain: at one extreme of the continuum, individual episodes are represented contextually in their spatial, temporal, and perceptual details, whereas at the other, common features of specific lifetime periods are thought to be represented in a more abstract format (Conway & Pleydell-Pearce, 2000; Renoult et al., 2012; D’Argembeau, 2020). Within this framework, PS has been theorized to situate on an intermediate position on this continuum, because it involves personal aspects that are less abstracted and more linked to specific episodes compared to general semantic knowledge (Renoult et al., 2012; Sheldon et al., 2020). Different categories of PS information have been further argued to differ in their position on the episodic-semantic continuum; some are more closely linked to their initial context of acquisition (Grilli & Verfaellie, 2014, 2016). In line with this conceptualization, more “experience-near” or “context-dependent” semantic information, such as repeated events, has been shown to prime more strongly the access to specific autobiographical episodes, compared with more abstract PS information (e.g., information on personality traits) and general semantic facts (Sheldon et al., 2020). Also, the retrieval of experience-near PS information (enPS), such as facts derived from repeated or specific events, but not that of more context-independent PS information (e.g., “I have a son”), has been shown to be impaired by medial temporal lobe lesions (Grilli & Verfaellie, 2014, 2016). Using intracranial recording, specific neural signatures in the posteromedial cortex have been found to be associated with the processing of autobiographical facts, self-knowledge, and specific events (Foster et al., 2012). Moreover, using EEG, repeated events and autobiographical facts have been differentiated from both specific events and general semantic knowledge; repeated events also could be more easily differentiated from general semantics than from specific events, supporting their close link to contextual elements (Renoult et al., 2016).

These finding are overall consistent with the proposal that a continuum between episodic and semantic memory can also be identified at the neural level, as both episodic and semantic memory rely on regions of the Default Mode Network (DMN) (Irish & Vatansaver, 2020). Evidence has been provided that regions of the DMN, including the posteromedial, medial prefrontal, inferior frontal cortex, the angular gyrus and the lateral and anterior middle temporal cortex, appear to have a strategical position in the unimodal-to-multimodal cortical processing hierarchy, allowing such regions to support mental representations ranging in specificity from concrete to highly abstract (Smallwood et al., 2021). Concerning the memory domain, it has been proposed that DMN nodes may interact with other brain regions in the context of macro-scale cortical gradients and that these interactions would determine the content of memory traces, allowing to account for the dynamic nature of AM representations, which typically involve both episodic and semantic information (Irish & Vatansaver, 2020). In this view, enPS may arise thanks to neural interactions that happen close to the episodic extreme of this continuum (Renoult et al., 2012; Sheldon et al., 2020).

Over the past 10 years, a growing body of literature has provided compelling evidence on the existence of relevant individual differences in autobiographical memory (see Palombo, Sheldon, & Levine, 2018, for a review). Such individual differences in behavior have been shown to be linked to specific variations in brain connectivity and anatomy. Studies investigating associations between individual differences in AM and anatomical variability have reported a positive association between the volume of the precuneus and the tendency to report EAMs from an egocentric perspective (Hebscher et al., 2018). Moreover, the number of internal details generated in an adapted version of the Autobiographical Interview (Levine et al., 2002)—a proxy of episodic autobiographical remembering—has been found to be positively associated with the volume of specific hippocampal subfields, namely the subiculum and the DG/CA2/3 in the left hemisphere (Palombo et al., 2018). Studies investigating structural connectivity also have provided insights on brain correlates of individual variations in EAM (Hodgetts et al., 2017; Williams et al., 2020). Fractional anisotropy (FA) in the inferior longitudinal fasciculus, linking occipital areas to the anterior temporal lobe, correlates with individual variations in the number of general semantic details recollected; FA in the fornix—and specifically in its precommissural portion, linking the hippocampus to the ventromedial prefrontal cortex (Williams et al., 2020)—correlates with the number of episodic autobiographical details (Hodgetts et al., 2017; Williams et al., 2020). More recently, Clark and colleagues (Clark et al., 2022) also reported an association between differences in the number of episodic autobiographical details recollected and the speed of conduction along the parahippocampal cingulum bundle, which connects the hippocampus with the parahippocampal, enthorinal, and retrosplenial cortex (Clark et al., 2022).

Other studies have provided evidence on the relation between AM variations in healthy participants and intrinsic functional connectivity using resting-state fMRI. Sheldon and colleagues (Sheldon et al., 2016) showed that individual variations in self-reported abilities in autobiographical episodic and general semantic memory correlate with the strength of connectivity of the medial temporal lobes, respectively with posterior, and inferior and middle prefrontal regions (Sheldon et al., 2016). Later studies showed that the degree of coupling between the DMN and the lateral prefrontal cortex predicts the quantity of nonepisodic details recollected in the Autobiographical Interview in older adults, suggesting an association with semanticization processes (Spreng et al., 2018). Similarly, stronger connectivity within the anterior portion of the posteromedial network has been associated with decreased production of episodic details only in older adults (Matijevic et al., 2022). Recently, the degree of reexperiencing of EAMs has been reported to be positively associated with the intrinsic connectivity of the temporal pole with the medial prefrontal and temporoparietal cortex, with that of the anterior hippocampus with the orbitofrontal and medial prefrontal cortex, and with that of the posterior hippocampus and parahippocampal cortex; the proportion of nonepisodic details, instead, was associated with the intrinsic connectivity of the temporal pole with temporoparietal and lateral temporal regions (Setton et al., 2022).

Overall, these results have provided converging evidence that individual differences in performance in tasks tapping EAM may map on structural variations mainly involving the medial temporal lobe (Palombo et al., 2018) and its anatomical connections (Hodgetts et al., 2017; Williams et al., 2020; Clark et al., 2022). Also, they strongly suggest that such individual differences in behavior are associated with specific patterns of intrinsic brain connectivity involving interactions between key nodes of the DMN with the rest of the brain (Spreng et al., 2018; Setton et al., 2022). However, whereas some of these studies also provided insights on brain correlates associated to variations in general semantic memory processing (Hodgetts et al., 2017; Sheldon et al., 2016), to the best of our knowledge no study has assessed brain correlates of individual differences in enPS. In the present study, we used resting-state fMRI to assess the relation between behavioral differences in episodic autobiographical and experience-near personal semantic memory, and patterns of brain connectivity. We assessed individual variations in the access to EAM and enPS in a sample of young individuals using an adapted version of the Autobiographical Fluency Task (AFT) (Dritschel et al., 1992). This test has been shown to effectively foster the strategic retrieval of EAMs and enPS, with cluster analysis suggesting that whereas both EAMs and enPS are generally separated from general semantics, autobiographical information tends to cluster together (Dritschel et al., 1992). The AFT has been previously used in different studies to assess autobiographical memory (Addis & Tippett, 2004; Tomadesso et al., 2015). This test shows good reliability and good convergent and divergent validity, as supported by the specific correlations between performance in the two conditions of the AFT and the autobiographical memory subscales of the Survey of Autobiographical Memory by Palombo and colleagues (Palombo et al., 2013) (Conti et al., 2023). Scores in the EAM condition of the AFT have been shown to be specifically correlated with the production of internal details in the Autobiographical Interview (AI) (Levine et al., 2002), whereas scores in the enPS condition correlate with the sum of both internal and external details in the AI (Conti et al., 2023). These findings support the specificity of the EAM condition to test episodic components of autobiographical memory, as well as the suitability of the enPS condition as a measure of context-dependent, experience-near personal semantics. The notion that the AFT effectively taps enPS also is in line with the results of a recent study, in which performance in both the EAM and enPS condition of the AFT was found to be reduced in cognitively unimpaired older adults at risk to develop Alzheimer’s disease (Grilli et al., 2021). Importantly, the AFT requires participants to access autobiographical memories from different life periods (see below); thus, this feature further allows to ensure that the retrieved EAMs and enPS are temporally contextualized and to consistently sample across individuals’ memories, providing a way to finely assess individual differences in the access to autobiographical memories.

According to the gradient perspective, EAMs and enPS may stem from interactions within the same networks (Irish & Vatansaver, 2020). We thus hypothesized that individual variations in enPS fluency may be associated with differences in the intrinsic connectivity of brain hubs pivotal for the recall of AMs that possess a spatial and temporal context, such as the HC and the posteromedial network (Setton et al., 2022; Ritchey & Cooper, 2020). To our knowledge, this is the first study to investigate the relationship between individual differences in the AFT and patterns of intrinsic connectivity. Thus, we used an intrinsic connectivity contrast (ICC) analysis (Martuzzi et al., 2011) to assess differences in connectivity associated with individual variations in EAMs and enPS fluency. The choice of such a data-driven approach based on graph theory allows to reduce possible bias due to the a priori selection of ROIs and to reveal the involvement of regions not identified by previous studies (Buckner et al., 2009; Martuzzi et al., 2011). The ICC was further combined with a secondary seed-based analysis (Martuzzi et al., 2011) to investigate connectivity both between and within networks.

## Materials and methods

### Participants

Thirty volunteers aged 23 to 33 years (mean age: 27.00 years, standard deviation [SD] = 2.573, 19 females) took part in the study. This sample partially overlapped with that included in a previous study from our group (Teghil et al., 2022). Sample size was in line with previous studies that assessed associations between resting-state functional connectivity and individual differences in cognition (Chong et al., 2017; Teghil et al., 2020; Deng et al., 2016). All participants had no previous or current history of neurological or psychiatric disorders, were right-handed, and had normal or corrected-to-normal vision. The study was designed in accordance with the principles of the Declaration of Helsinki and approved by the ethical committee of IRCCS Fondazione Santa Lucia, Rome. Informed consent was obtained from all individual participants.

### Autobiographical fluency task

Individual differences in the access to EAM and PS were assessed using an adapted version of the Autobiographical Fluency Task (AFT) (Dritschel et al., 1992), performed outside the scanner.

For each of five life periods, participants were asked to report personal events occurred to them at a specific time and place (EAMs) and names of personally known people (e.g. friends, teachers, schoolmates, colleagues) that they have met during that period and were specifically associated with that, but not other periods (enPS). Probed life periods were 5–11 years, 11–14 years, 14–19 years, >19 years excluding the last 12 months, and the period corresponding to the past 12 months. For each combination of period and category (EAM and enPS), participants were asked to report as many items as possible within a 90-s time limit, without further elaborating on the retrieved items.

In the EAM condition, participants were asked to report labels corresponding to their personal events; it was specified that they could choose any label that was meaningful to them and that allowed them to clearly identify the event. Instructions emphasized that for the EAM condition, participants had to report events that happened at a specific time and place, and an example was provided to facilitate compliance with task instructions (the experimenter stated that if they wanted to recall the time they visited an impressionist painters exhibition at the Vittoriano in Rome in 2016, they could provide a label such as “impressionist painters’ exhibition”).

Concerning the enPS condition, participants also were told that they should not report names of individuals associated with more than one life period (e.g., names of parents or siblings). This ensured that responses were actually derived from specific lifetime period, according to the definition of enPS by Grilli and Verfaellie (2016). Also, participants were told that they did not have to provide surnames for the named people, but rather could simply provide the initial letter of surnames in order to disambiguate between individuals with the same first name. Whenever the examiner was in doubt concerning the presence of repetitions, doubts were cleared up with the participant. In case a participant named the same individual for more than one life period, at the end of the 90-s period, he or she was reminded that it was not possible to provide the name of the same person for multiple periods and was asked to choose only one period in which the name should be collocated. For the EAM condition, whenever the examiner was in doubt that a participant recalled a repeated rather than a unique event, this was checked out with the participant at the end of the 90-s period, and the participant was reminded that events should be collocated in a specific time and place. All repeated events were excluded.

To ensure compliance with instructions, at the end of task administration, participants were asked to report their age at the time of each specific event in the EAM condition, as well as their age when they first met each person named in the enPS condition. For each category, a fluency score was calculated as the sum of the individual items reported by the participant. All repetitions were excluded from response count.

### Image acquisition

Magnetic resonance images were acquired on a 3-T scanner (Siemens MAGNETOM Prisma), equipped with a 32-channel head coil. Two resting-state fMRI scans were acquired for each participant using a T2٭-weighted, gradient-echo echo-planar imaging (EPI) sequence, a multiband factor of 4, and an isotropic voxel size of 2.4 mm^3^ (60 slices, field of view [FOV] 208 x 208 mm^2^, repetition time [TR] = 1100 ms, echo time [TE] = 30 ms, flip angle = 65°, no in-plane acceleration) (Moeller et al., 2010; Feinberg et al., 2010; Xu et al., 2013). Three hundred fMRI volumes were acquired in each run, including four dummy scans before each run, which were discarded. Two spin-echo EPI volumes with phase encoding in opposite direction, no multiband acceleration, and the same geometrical and sampling properties of functional runs were acquired for field mapping (TE = 80 ms, TR = 7000 ms). T1-weighted structural images were acquired for each participant using an MPRAGE sequence (Hess et al., 2011; Tisdall et al., 2012). Volumetric imaging included 176 slices, isotropic resolution 1 mm^3^, TR = 2500 ms, TE = 2 ms, inversion time [IT] = 1070 ms, flip angle = 8°. During resting-state fMRI scans, participants were asked to lay at rest with eyes closed and not to fall asleep.

## Analysis of imaging data

### Preprocessing

Resting-state fMRI data were analyzed using the CONN toolbox (v. 20b) (Whitfield-Gabrieli & Nieto-Castanon, 2012; http://www.nitrc.org/ projects/conn). A field map was computed from the spin-echo EPI images acquired with opposite encoding polarity (Holland et al., 2010). After removal of the first four scans, functional images were corrected for head movements and B0-distortion, including motion x field interaction (realignment and unwarping, Andersson et al., 2001) by using the first volume as reference and resampled to a voxel size of 2 x 2 x 2 mm^3^. Time series were interpolated to correct for slice-timing distortions. Structural images were segmented in gray matter, white matter (WM), and cerebrospinal fluid (CSF) for successive use during removal of temporal confounding factors and normalized to MNI space. After normalization, ART-based scrubbing (Power et al., 2012) was applied. Functional data were smoothed by using a 6-mm^3^, full-width, half-maximum (FWHM) Gaussian kernel. Temporal confounding factors (time courses of WM and CSF BOLD signals, a linear trend, and the six motion parameters derived from the previous realignment procedure) were removed from the BOLD time series of functional data, regressing them out at each voxel. A band-pass filter (0.008–0.09 Hz) was applied to resulting residual time series.

### Intrinsic connectivity contrast analysis

We performed an intrinsic connectivity contrast (ICC) analysis to characterize network differences associated with the EAM and enPS conditions. ICC is a whole-brain, voxel-based measure of connectivity based on network theory that allows to define how strongly any given voxel is connected to the rest of the voxels in the brain, without specifying any a priori ROI (Martuzzi et al., 2011). Thus, ICC represents a measure of node centrality, that is the strength of the connectivity between a voxel and the rest of the brain. ICC takes into account both the presence and the strength of connections and allows to calculate voxel-level maps that reflect the intrinsic connectivity contrast of individual voxels; it does not require a priori assumptions and provides a global picture of the effect of a specific condition on local connectivity patterns (Martuzzi et al., 2011). ICC was calculated as the root mean square of the correlation coefficients between each voxel and all voxels in the brain (Martuzzi et al., 2011), according to the procedure implemented in CONN (Whitfield-Gabrieli & Nieto-Castanon, 2012).

Intrinsic connectivity contrast maps were calculated respectively for the EAM and enPS conditions of the AFT. More in detail, we calculated ICC specifically associated with performance in the EAM and enPS conditions entering respectively EAM and enPS scores in two multiple regression models at the second level analysis, while controlling for performance in the other condition. This allowed us to characterize brain regions with which ICC patterns are specifically associated with individual differences in the EAM and enPS conditions of the AFT, controlling for confounding factors associated with general proficiency in autobiographical fluency (see below). A voxel threshold of *p* < 0.001 uncorrected and a cluster-size p-FDR corrected of *p* < 0.05 (one-tailed positive) were used for this analysis.

### Seed-to-voxel analysis

To identify which connections of the nodes identified with the ICC analysis were affected by the experimental condition (EAM vs. enPS), we entered the regions identified in the ICC analyses as seed regions in a seed-to-voxel analysis. Specifically, for each seed, we entered performance in the enPS condition of the AFT in the multiple regression model at the second level analysis, while controlling for performance in the EAM condition (the opposite model, namely the model testing the effect of the EAM condition while controlling for performance in the enPS condition, was not tested since the ICC analysis did not result in any significant suprathreshold voxel; see the *Results* section below). A voxel threshold of *p* < 0.001 uncorrected and a cluster-size p-FDR corrected of *p* < 0.025 (one-tailed positive) was used for this analysis.

### ROI-to-ROI analyses within autobiographical memory networks

To characterize more fully the intrinsic connectivity patterns associated with individual variations in performance in the EAM and enPS conditions of the AFT, we performed an additional analysis investigating the association between scores in the two conditions, and the strength of the functional coupling between a set of brain regions defined a priori, corresponding to regions of the DMN supporting autobiographical memory based on previous fMRI studies (Teghil et al., 2021). Full methods and results are reported in Supplementary Materials.

## Results

### Performance in the AFT

Mean scores were respectively 35.633 (standard deviation [SD] = 7.815) and 50.267 (SD = 12.415) in the EAM and enPS conditions of the AFT. A paired-sample *t*-test showed that the difference between performance in the two conditions was statistically significant (t(29) = −8.986, *p* < 0.001). A two-tailed correlation analysis was also performed between the two conditions of the AFT. Because the two conditions were found to be moderately-to highly correlated (r = 0.699, *p* < 0.001), all associations between resting-state connectivity and scores in the EAM and enPS conditions were investigated entering score in the other condition as a control variable (see above). Following the original study by Dritschel and colleagues (1992), we further calculated a similarity index (SI) between the two conditions of the AFT in each lifetime periods. Participants’ scores were standardized to range 0–100; the mean absolute difference between the standardized scores in the two conditions was calculated for each participant, and these indices were subtracted from 100 to derive similarity indices (Dritschel et al., 1992). Results showed that similarity indices were overall high (period 5–11: M = 69.00, SD = 20.75; period 11–14: M = 77.11, SD = 19.07; period 14–19: M = 76.70, SD = 15.81; period 19–last year: M = 79.86, SD = 18.94; last year period: M = 79.18, SD = 15.02), again suggesting the presence of a correlation between fluency in the two conditions of the AFT for the different lifetime periods. A repeated-measures ANOVA performed on SI in the different lifetime periods showed that similarity in fluency in the two conditions of the AFT was not significantly different across periods (F(4,120) = 1.935, *p* = 0.109, η_p_^2^).

### ICC analysis

**Fig. 1:**
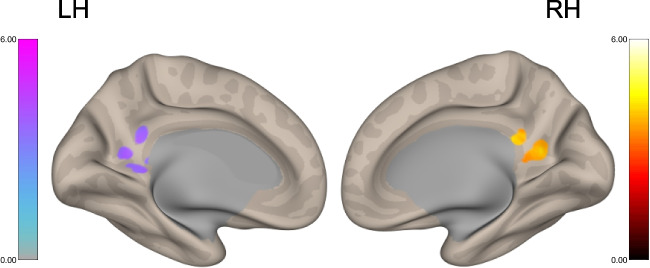
Results of the ICC analysis performed entering enPS scores in a regression model at the second level analysis, controlling for performance in the EAM condition. RH = right hemisphere; LH = left hemisphere

The results of the ICC analysis showed two clusters in the right and left posterior cingulate cortex (PCC) (right: MNI +10, −48, +18, 99 voxels, t (27) = 4.93, cluster-size p-FDR corrected = 0.011, Cohen’s d = 0.95; left: MNI −10, −52, +14, 72 voxels, t (27) = 5.10, cluster-size p-FDR corrected = 0.024, Cohen’s d = 0.98), which connectivity with the rest of the brain was positively associated with performance in the enPS condition of the AFT (Fig. 1). No cluster was found to be significantly associated with scores in the EAM condition.

### Seed-to-voxel analysis

**Fig. 2:**
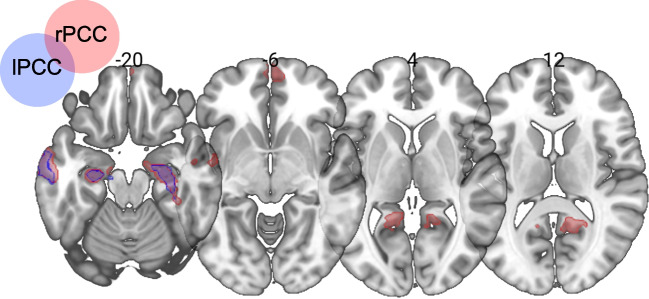
Results of the seed-to-voxel analyses performed entering regions identified in the ICC analyses as seed regions and performance in the enPS condition of the AFT in the multiple regression model at the second level analysis, while controlling for performance in the EAM condition. Results for the right and left PCC seeds are respectively shown in light red and light blue.

Results of the seed-to-voxel analysis are reported in Table 1. Seed-to-voxel analyses performed on regions highlighted by the ICC analysis showed that the connectivity between both seeds and different clusters of voxels was associated positively with scores in the enPS condition of the AFT after controlling for performance in the EAM condition. Specifically, enPS fluency scores predicted the strength of the connectivity of the right PCC seed with clusters in several brain regions, including the bilateral anterior hippocampus (HC), precuneus (pCu), anterior middle temporal gyrus (aMTG), and medial orbitofrontal cortex (mOFC) in the right hemisphere and the aMTG and PCC extending to the lingual gyrus (LG) in the left hemisphere (Fig. 2). enPS fluency scores also predicted the strength of the intrinsic connectivity of the left PCC seed with clusters of voxels in the bilateral anterior HC and MTG (Fig. 2).

**Table 1:** Significant results (cluster-size p-FDR < 0.025) of the seed-to-voxel analyses using ROIs derived from the ICC analysis as seeds. For each significant cluster MNI coordinates, size and corrected and uncorrected p are reported. PCC = posterior cingulate cortex; HC = hippocampus; MTG = middle temporal gyrus; pCu = precuneus; mOFC = medial orbitofrontal cortex; LG = lingual gyrus; MTG = middle temporal gyrus; r = right hemisphere; l = left hemisphere; a = anterior

Seed	Region	MNI (x, y, z)			Cluster size	Cluster-size p-FDR	Cluster-size p-unc
rPCC	raHC	10	−12	−16	410	0.000008	<0.00000
	laMTG	−64	−4	−20	403	0.000008	<0.00000
	rpCu	12	−54	12	385	0.000008	<0.00000
	rmOFC	4	60	−6	282	0.000082	0.000007
	raMTG	64	0	−28	280	0.000082	0.000007
	lHC	−28	−16	−22	231	0.000282	0.000031
	lPCC/LG	−16	−50	4	140	0.004614	0.000587
lPCC	raHC	26	−14	−20	500	0.000001	<0.00000
	lMTG	−64	−14	−24	406	0.000004	<0.00000
	laHC	−22	−20	−14	194	0.001182	0.000081
	rMTG	56	−8	−30	179	0.00145	0.000132

## Discussion

We used individual differences in behavior as a model to investigate brain networks associated with the access to EAM and enPS information. To this goal, we assessed the relation between performance in a fluency task requiring to produce all possible EAMs and enPS for specific lifetime periods and intrinsic brain connectivity, in line with previous evidence that interindividual variations in AM can be reliably associated with differences in resting-state connectivity profiles (Sheldon et al., 2016; Spreng et al., 2018; Matijevic et al., 2022; Setton et al., 2022).

First, the results of the ICC analysis highlighted different effects of the two task conditions (EAM and enPS) on local patterns of intrinsic connectivity. While no brain region showed ICC patterns associated with individual variations in the EAM condition of the AFT, we found that the global connectivity of two clusters in the left and right PCC was significantly predicted by performance differences in the enPS condition.

Activation of the PCC has been consistently reported by task-based neuroimaging studies on AM, involving both episodic and semantic AM tasks (Spreng et al., 2009; Araujo et al., 2013; Martinelli et al., 2013; Teghil et al., 2021, for meta-analyses). More in detail, activation of the PCC specifically during EAM and enPS tasks was found in a previous meta-analysis using activation likelihood estimation, suggesting that a posterior-to-anterior gradient of activation may correspond to an increase in the level of abstraction of AMs (Martinelli et al., 2013). Activation of the PCC has also been reported to be associated with the retrieval of conceptual knowledge about autobiographical events (Gurguryan & Sheldon, 2019), and this region has been more generally proposed to be a key node within a network supporting the development and use of situation models, conceived as schematic representation of temporal, spatial, and causal relations that apply within specific contexts (Ranganath & Ritchey, 2012). Accordingly, the retrosplenial complex, including the PCC and RSC, as well as the aMTG and the vmPFC, have been found to be activated when participants were asked to temporally order enPS information across different lifetime periods (Teghil et al., 2022), supporting the possibility that the retrosplenial complex may be involved in the representation of temporal relations in the autobiographical domain.

The PCC is considered a key node of the DMN, together with the anterior medial prefrontal cortex; these two regions have been proposed to form a midline “core” subsystem within the DMN, which fluctuations at rest are more highly correlated with those of the others DMN regions (Andrews-Hanna et al., 2010, 2014). This is in line with the results of our second set of analyses, in which regions highlighted by the ICC analysis were separately entered in two seed-to-voxel analyses, allowing to further characterize networks associated with individual performance in the enPS condition of the AFT. Results of these latter analyses showed a relation between variations in autobiographical fluency for enPS and patterns of intrinsic connectivity of the two PCC seeds with a broad neural network, commonly involving the bilateral aHC and aMTG. Also, an association was found between the strength of the connectivity of the right PCC with the ipsilateral medial frontal cortex and pCU and the left PCC/lingual gyrus. All of these regions are known to be part of the DMN (Andrews-Hanna et al., 2010, 2014); specifically, whereas the medial prefrontal cortex and the PCC map on the “core” network of the DMN (DMN-A), the lateral temporal and ventral prefrontal cortex are considered part of the dorsal-medial subnetwork (DMN-B) (Yeo et al., 2011; Baker et al., 2014). These two cortical subsystems of the DMN have been proposed to access and process different types of self-generated information. Whereas previous research has consistently associated the core network of the DMN with self-related processing, including self-referencing, personal significance, and autobiographical memory (Andrews-Hanna et al., 2014; Davey et al., 2016), the dorsal-medial subsystem appears to be involved in processing stored thematic, conceptual, and schematic information related to a person’s experiences (Andrews-Hanna et al., 2014; Andrews-Hanna & Grilli, 2021). Also, regions of the lateral temporal cortex have been specifically implicated in the abstract representation of autobiographical information, including temporal schemas, such as lifetime periods (D’Argembeau, 2020). In the present study, we also found an association between individual variations in the access to enPS and the strength of connectivity between the bilateral PCC seeds and the anterior portion of the HC. The aHC has been implicated in the retrieval of generalized autobiographical information and appears to be oriented toward the construction of abstracted AM representations (Sheldon & Levine, 2016; Sheldon et al., 2019). In line with the continuum perspective, it has been proposed that the HC may contribute to the construction of AM representations with different levels of specificity, according to task demands (Sheldon et al., 2019). The present results, showing that individual variations in the access to enPS are associated with patterns of connectivity within a network involving regions of both the core and dorsal-medial subnetworks and the aHC, are in line with the possibility that enPS may represent a peculiar entity within the episodic-to-semantic continuum, entailing both self-relevance and a certain degree of abstraction compared with EAM (Renoult et al., 2012; Grilli & Verfaellie, 2014).

It is worth noting that the network highlighted in the present study partially overlaps with the so-called “posterior medial” (PM) network, which has been implicated in episodic retrieval (Ranganath & Ritchey, 2012; Libby et al., 2012; Ritchey & Cooper, 2020). Within the PM network, separate subnetworks of brain regions have been proposed to form “core alliances” to support different kind of representations, including AM (Ritchey & Cooper, 2020). Specifically, the authors proposed that interactions of the HC with regions, such as the parahippocampal cortex, the precuneus, and the angular gyrus, could be more associated with the processing of visuospatial and contextual features and thus would allow to organize events within a contextual framework also accounting for imaginal features of EAM. Other regions, such as the aHC, mPFC, and PCC, could be more strongly linked to the representation of schematic and gist-like features of experience (Ritchey & Cooper, 2020). The present results suggest the intriguing possibility that a subset of regions within the PM network may actually interact dynamically to support the retrieval of enPS representations, which involve schema knowledge and a gist-like representation of common features of events but do not require mental imagery or detailed episodic retrieval. Within this framework, functional interactions within DMN regions could arise in response to task demands (Cabeza et al., 2018), allowing to construct AM representations ranging in the degree of specificity; such task-relevant interactions would in turn be reflected in inter-individual variations in patterns of connectivity at rest. Future studies are warranted to test this hypothesis directly and, more generally, to characterize in detail network interactions accounting for the specific features of different formats of retrieved autobiographical knowledge.

Finally, we did not find any association between ICC patterns and individual differences in the access to EAMs. This finding may look at odds with previous literature, showing a relationship between interindividual variations in autobiographic recollection and connectivity patterns of the medial temporal lobe with medial prefrontal and posterior cortical regions (Sheldon et al., 2016; Setton et al., 2022). Although negative findings should be interpreted with caution, whereas previous studies investigating intrinsic connectivity patterns associated with variations in EAM often used interviews designed to elicit a detailed recollection of specific life events (Setton et al., 2022; Matijevic et al., 2022; Spreng et al., 2018), we assessed differences in AM using the AFT, requiring to produce as many items as possible for each memory category (Dritschel et al., 1992; Conti et al., 2023). Thus, this test did not require participants to fully recollect episodes in the EAM condition. It is possible that, given the rich multisensorial qualities of EAMs, other types of instruments, such as tests requiring a detailed recollection, may better characterize subtle individual variations in EAM performance in healthy, young individuals. It also has been shown that retrieving specific personal events may produce more complex brain responses compared with retrieving general semantic information, possibly due to the former entailing the re-instantiation of different features of episodic information (Heisz et al., 2013). Because variable features of an EAM could be more easily retrieved according to individual differences in process components underlying autobiographical memory (Palombo, Sheldon, & Levine, 2018), this could result in a more complex neural signature for EAM, reducing possibilities to detect a consistent association between intrinsic network connectivity and behavioral performance. Conversely, in line with our previous findings that enPS appears to rely on thematic relations supported by schema-knowledge (Teghil et al., 2022), individual variations in fluency in this dimension may map on a more distributed mode of information processing, allowing the representation of more highly redundant information, abstracted across multiple experiences (Heisz et al., 2013). This mechanism would fit well with the notion of *schema* proposed by Squire and colleagues (2015), as well as with the existence of complementary learning systems (McClelland et al., 1995; Kumaran et al., 2016), and in particular with the neocortical processing system, namely the temporally extended system level consolidation, which spans for years or decades, is derived from repeated exposure to events and allows the acquisition of stable schema-consistent information in the neocortex (Kumaran et al., 2016). Such interpretations, however, are speculative at present. Further research would be needed to test these possibilities.

The present study has different limitations. As mentioned, because the AFT does not require to elaborate on retrieved items, this test mainly assesses individual variations in the strategic retrieval of autobiographical memories (Grilli et al., 2021). Thus, present results are not directly informative of connectivity patterns associated with complete access and elaboration of such memories. An important direction for future research will be to assess specific neural mechanisms differentially supporting EAM and enPS using methodologies allowing to investigate both the access, retrieval, and elaboration of different formats of autobiographical representations.

It has to be pointed out that no narrative or full description of recalled memories was required in this study. As such, the degree of episodic specificity of items produced in the EAM conditions could not be objectively verified (e.g., with an independent coding of provided narratives, see Hitchcock et al., 2020). This point is specifically important given that component processes involved in the AFT have not been completely investigated to date. The original definition of “experience-near personal semantics” refers to personal knowledge that is more associated with spatiotemporal details in comparison with predominantly conceptual and abstract types of personal semantic knowledge (Grilli & Verfaellie, 2014, 2016). Nonetheless, this definition encompasses different categories of personal semantic information, including knowledge derived from lifetime periods, repeated events, and specific events (Grilli & Verfaellie, 2016). Because the enPS condition of the AFT requires naming individuals associated with specific time periods, it appears more well suited to assess knowledge associated with lifetime periods or repeated events, rather than derived from specific events. Moreover, it has been highlighted that even the retrieval of categories of personal semantics considered to be more abstract, such as self-knowledge of personality traits, may interact with episodic processes, determining different brain signatures depending on specific task demands (Tanguay et al., 2018, 2020). Thus, connectivity patterns highlighted in the present study are likely not unique and invariable neural signatures associated with the access to experience-near personal semantic information.

Task-based fMRI studies have shown that formats of autobiographical representations (e.g., episodic autobiographical memories, autobiographical facts) and general semantics may involve activation within a common network, including the medial frontal cortex, the superior and middle temporal gyrus, the temporoparietal junction, the precuneus, the left hippocampus, the parahippocampal gyrus, and the frontal pole (Maguire & Frith, 2003; Holland et al., 2011; Tanguay et al., 2023), suggesting that these different formats, as well as their phenomenological properties, may correspond to incremental levels of neural activity within a shared network. The AFT used in this study specifically assessed a category of experience-near personal semantics, associated with different life periods. Thus, although present findings shed light on brain networks associated with individual differences in the access to this type of enPS, not completely overlapping patterns may underlie individual variations in the access to other categories of personal semantic information, as well as individual differences in different phases of the retrieval process (e.g., construction of retrieved memories).

Because this was the very first study to assess brain correlates of individual differences in the access to experience-near personal semantics versus episodic autobiographical memories, we choose to use the AFT, which has been previously shown to be a reliable and effective measure of autographical memory (Dritschel et al., 1992; Addis & Tippett, 2004; Tomadesso et al., 2015; Grilli et al., 2021; Conti et al., 2023). Future studies should extend the present investigation to different types of personal semantic information to establish more reliably a correspondence between individual differences in these domains and intrinsic connectivity patterns. Furthermore, it will be of key importance to assess more deeply the specificity of the recollected items for the EAM and enPS condition of the AFT, also using specific scoring systems (e.g., the revised Autobiographical Interview scoring system developed by Renoult and colleagues (Renoult et al., 2020) to allow a more complete characterization of component processes involved in this task.

Finally, it is being increasingly acknowledged that the investigation of brain-behavior associations in very large dataset is desirable to ensure reproducibility (e.g., Marek et al., 2022). This issue is particularly relevant in memory studies, because strategies used to perform fluency tasks may significantly impact on involved brain networks (Greenberg et al., 2009; Sheldon & Moscovitch, 2012). Future research should replicate the present results using larger samples, possibly also assessing putative individual variations in retrieval and search strategy.

## Conclusions

Present results provide insights on specific neural dynamics supporting the retrieval of enPS and are consistent with proposals that AM representations with different levels of specificity may map onto a neural continuum, supported by DMN regions (Irish & Vatansaver, 2020). In this light, interaction allowing the instantiations of enPS may entail some, but not all, component processes required by EAMs, thus relying on DMN regions that support both the retrieval of conceptual autobiographical knowledge, and the attribution of self-relevance, while not involving brain regions supporting mental imagery and egocentric perspective taking, which are typically associated with EAM (Hebscher et al., 2018, 2020).

### Supplementary Information

Below is the link to the electronic supplementary material.

Supplementary file1 (PDF 6735 KB)

## Data Availability

The conditions of our ethics approval do not permit public archiving of the raw MRI data. The preprocessed MRI anonymous data are available at the corresponding author on reasonable request.
