# Supplementary material for: Brain connectivity patterns associated with individual differences in the access to experience-near personal semantics: a resting-state fMRI study
Source: Cogn Affect Behav Neurosci. 2024 Jan 10;24(1):87–99. doi: 10.3758/s13415-023-01149-6 (PMC10827898; doi:10.3758/s13415-023-01149-6)
Supplement: Supplementary file 1 — Supplementary file1 (PDF 6735 KB) [file 13415_2023_1149_MOESM1_ESM.pdf]

Supplementary materials

ROI-to-ROI analyses within autobiographical memory networks

Method

This set of analyses assessed the association between scores in the EAM and enPS conditions of the AFT, and variations in resting-state connectivity between regions of the DMN supporting autobiographical memory according to previous fMRI studies. To this aim, we defined 9 spherical ROIs (10-mm radius) centered on peak coordinates derived from an activation likelihood estimation meta-analysis on autobiographical memory (Teghil al., 2021). Defined ROIs included: left ventromedial prefrontal cortex (vmPFC) (center at MNI -4, 50, -6), right (center at MNI: 52, -68, 32) and left (center at MNI: -46, -68, 32) angular gyrus (AG), right (center at MNI: 10, -50, 32) and left (center at MNI: -8, -54, 14) posterior cingulate cortex (PCC), right (center at MNI: 26, -14, -18) and left (center at MNI: -24, -26, -16) hippocampus (HC), right (center at MNI: 58, -4, -18) and left (center at MNI: -58, -4, -22) anterior middle temporal gyrus (aMTG).

In order to assess connectivity patterns associated with individual differences in the AFT, independently from the specific task condition, we performed an F-test to assess any effect among the EAM and enPS conditions on connectivity between the ROIs mentioned above. Results were thresholded using Spatial Pairwise Clustering (Zalesky et al., 2012), with a cluster-level p-FDR corrected < 0.05.

Then, for each pair of ROIs, we assessed the association between Pearson's correlations between the two corresponding regional BOLD time-courses, and scores in the two conditions of the AFT (EAM and enPS). Participants' scores in the two conditions were entered in two multiple regression models at the second level. Notably, in order to more fully characterize connectivity patterns associated with individual variations in the EAM and enPS condition of the AFT task, this analysis was performed without controlling for performance in the other condition. Results were thresholded using Spatial Pairwise Clustering (Zalesky et al., 2012), with a cluster-level p-FDR corrected < 0.05 (one-tailed positive).

Results

Results of the F-test on any effect among the EAM and enPS condition are reported in Table S1 and shown in Figure S1.

**Analysis Unit**	**Statistic**	**p-unc**	**p-FDR**
Cluster 1/3	Mass = 76.01	0.001974	0.005922
Connection rHC-lPCC	F(2,27) = 16.60	0.000020	0.000717
Connection lHC-lPCC	F(2,27) = 7.67	0.002302	0.031229
Connection rHC-lvmPFC	F(2,27) = 7.06	0.003419	0.031229
Connection lHC-lvmPFC	F(2,27) = 6.67	0.004411	0.03176

Table S1. Results of the ROI-to-ROI analyses within autobiographical memory networks. Clusters of connections showing any effect among the EAM and enPS conditions of the AFT.

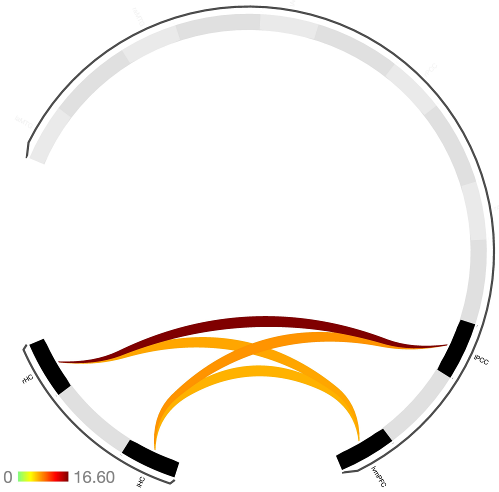

Figure S1. Clusters of connections showing any significant associations with scores in the EAM and enPS conditions of the AFT.

Results for EAM and enPS conditions are reported in Table S2 and shown in Figures S2 and S3.

Condition	Analysis Unit	Statistic	p-unc	p-FDR
EAM	*Cluster 1/1*	Mass = 31.35	0.036833	0.036833
	Connection raMTG-laMT	T(28) = 3.01	0.002769	0.099666
	Connection lAG-raMTG	T(28) = 2.58	0.007771	0.139881
enPS	*Cluster 1/5*	Mass = 77.76	0.003361	0.016806
	Connection rHC-lPCC	T(28) = 3.43	0.000945	0.017016
	Connection lHC-lvmPFC	T(28) = 3.26	0.001476	0.017115
	Connection lHC-lPCC	T(28) = 3.01	0.002708	0.017115
	Connection rHC-lvmPFC	T(28) = 2.73	0.005478	0.024652
	Cluster 2/5	Mass = 35.36	0.014146	0.035365
	Connection rHC-laMTG	T(28) = 2.98	0.002953	0.017115
	Connection rHC-raMTG	T(28) = 2.97	0.003052	0.017115
	Cluster 3/5	Mass = 30.25	0.021333	0.035556
	Connection lPCC-laMTG	T(28) = 2.93	0.003328	0.017115
	Connection lPCC-raMTG	T(28) = 2.56	0.008157	0.029364
	Cluster 4/5	Mass = 26.94	0.032683	0.040854
	Connection raMTG-laMTG	T(28) = 3.67	0.000505	0.017016

Table S2. Results of the ROI-to-ROI analyses within autobiographical memory networks. Clusters of connections showing significant associations with scores in the EAM and enPS conditions of the AFT.

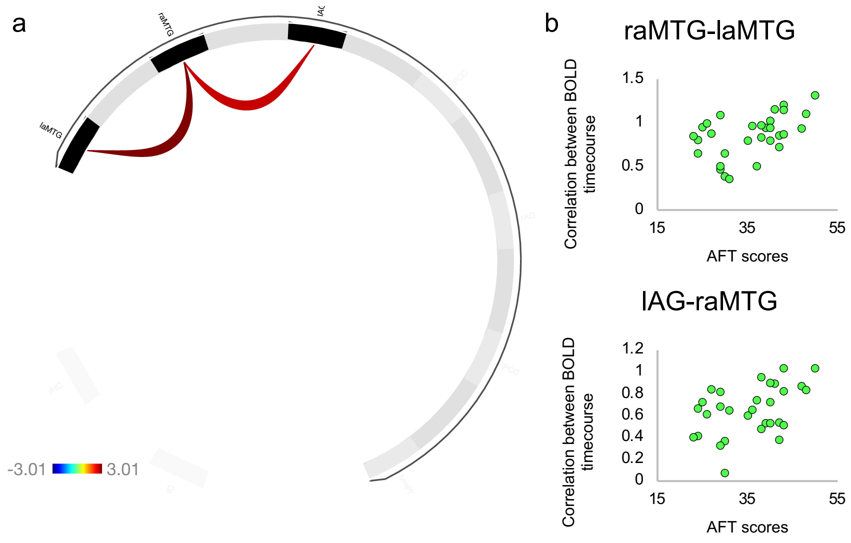

Figure S2. a) Clusters of connections showing significant associations with scores in the EAM condition of the AFT. b) Scatterplots of the correlation between scores in the EAM condition of the AFT and Fisher's r-to-z transformed values of the correlation between BOLD timecourse in pairs of ROIs.

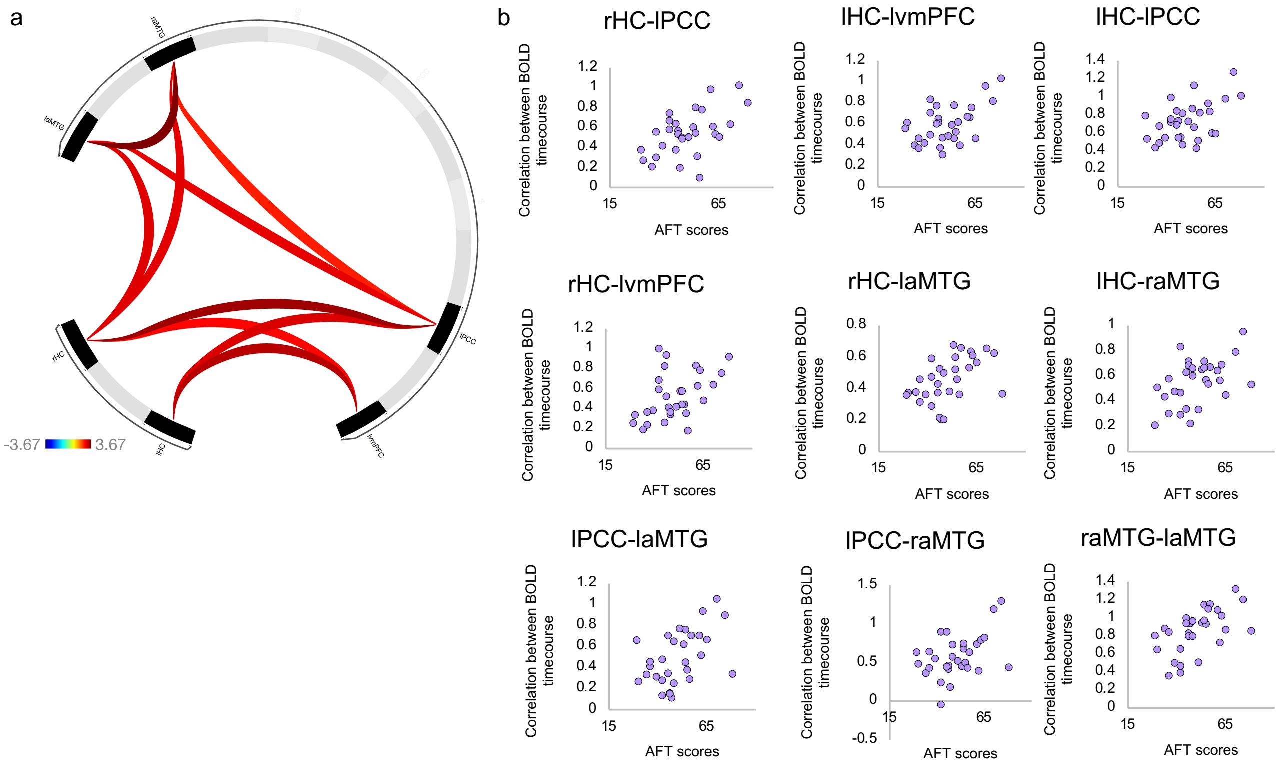

Figure S3. a) Clusters of connections showing significant associations with scores in the enPS condition of the AFT. b) Scatterplots of the correlation between scores in the enPS condition of the AFT and Fisher's r-to-z transformed values of the correlation between BOLD timecourse in pairs of ROIs.

References

Teghil, A., Bonavita, A., Guariglia, C., & Boccia, M. (2021). Commonalities and specificities between environmental navigation and autobiographical memory: A synthesis and a theoretical perspective. Neuroscience and biobehavioral reviews, 127, 928–945. https://doi.org/10.1016/j.neubiorev.2021.06.012

Zalesky, A., Fornito, A., & Bullmore, E. T. (2010). Network-based statistic: identifying differences in brain networks. Neuroimage, 53(4), 1197-1207.
